# Using Adipose Measures from Health Care Provider-Based Imaging Data for Discovery

**DOI:** 10.1155/2018/3253096

**Published:** 2018-09-27

**Authors:** Elliot D. K. Cha, Yogasudha Veturi, Chirag Agarwal, Aalpen Patel, Mohammad R. Arbabshirani, Sarah A. Pendergrass

**Affiliations:** ^1^Biomedical and Translational Informatics Institute, Geisinger Research, Danville, PA, USA; ^2^Department of Imaging Science and Innovation, Geisinger Research, Danville, PA, USA; ^3^Department of Electrical & Computer Engineering, University of Illinois at Chicago, Chicago, IL, USA; ^4^Department of Radiology, Geisinger, Danville, PA, USA

## Abstract

The location and type of adipose tissue is an important factor in metabolic syndrome. A database of picture archiving and communication system (PACS) derived abdominal computerized tomography (CT) images from a large health care provider, Geisinger, was used for large-scale research of the relationship of volume of subcutaneous adipose tissue (SAT) and visceral adipose tissue (VAT) with obesity-related diseases and clinical laboratory measures. Using a “greedy snake” algorithm and 2,545 CT images from the Geisinger PACS, we measured levels of VAT, SAT, total adipose tissue (TAT), and adipose ratio volumes. Sex-combined and sex-stratified association testing was done between adipose measures and 1,233 disease diagnoses and 37 clinical laboratory measures. A genome-wide association study (GWAS) for adipose measures was also performed. SAT was strongly associated with obesity and morbid obesity. VAT levels were strongly associated with type 2 diabetes-related diagnoses (*p* = 1.5 × 10^−58^), obstructive sleep apnea (*p* = 7.7 × 10^−37^), high-density lipoprotein (HDL) levels (*p* = 1.42 × 10^−36^), triglyceride levels (*p* = 1.44 × 10^−43^), and white blood cell (WBC) counts (*p* = 7.37 × 10^−9^). Sex-stratified tests revealed stronger associations among women, indicating the increased influence of VAT on obesity-related disease outcomes particularly among women. The GWAS identified some suggestive associations. This study supports the utility of pursuing future clinical and genetic discoveries with existing imaging data-derived adipose tissue measures deployed at a larger scale.

## 1. Introduction

The known relationship between the level of abdominal adipose tissue and metabolic syndrome and cardiovascular diseases (CVDs) is longstanding, including the risk for type 2 diabetes and sleep apnea. The developed world's adult population is showing ever-increasing rates of obesity and, consequently, an increase in a wide range of health risks [1]. Developing a deeper understanding of the impact of obesity on health risk using more detailed quantitative traits of obesity, beyond body mass index (BMI), such as different types of adipose tissue, can provide more insights into health risks and the biology of the impact of obesity.

Although BMI has been used for a wide range of research including genetic epidemiology, BMI has a distinct limitation in that the measurements assume a uniform contribution to risk by all adipose tissue, not taking into account the variation of the adiposity type and location from individual to individual. Location of adipose tissue has a critical role in the overall impact of obesity, with centralized adiposity having a higher impact on metabolic disorder health risks [2]. Furthermore, adipose is not a single homogeneous tissue and has regional deposits of both subcutaneous adipose tissue (SAT) and visceral adipose tissue (VAT) [3]. While SAT lies directly under the skin, VAT is the adipose tissue around the organs. Past epidemiological studies have suggested that VAT is more strongly associated with CVD and metabolic syndrome [4].

Other measures of adiposity such as waist-hip ratio (WHR) have proved to be predictive measures of diabetes risk in men [5] and coronary heart disease risk in both sexes [6, 7]. While WHR is appropriate for determining the regional distribution of adipose tissue [8], it has shown moderate associations with the amount of VAT accumulation in the abdomen [9–11] and large variability in distinguishing VAT from SAT [8]. Waist circumference measures have shown stronger associations with abdominal VAT compared to WHR in both men and women [8, 11–13].

Alternatively, the levels of VAT and SAT can be more accurately measured through computed tomography (CT), ultrasound, and magnetic resonance imaging (MRI) [14]. Research on the impact of obesity using CT scans has provided valuable insights into the risk of CVD [15]. Additionally, CT measures of adipose tissue have facilitated the exploration of the relationship between accumulation of abdominal SAT or VAT and genetic variation, showing sex-specific loci associated with VAT levels [16, 17]. Although there are known genetic contributions to adipose distribution [18–21], identifying genetic biomarkers using quantitative adipose data from large-scale CT studies provides an opportunity for a better understanding of the nexus between genetics, adiposity, and an exhaustive list of health outcomes and laboratory measures.

An obstacle to large-scale imaging analyses is the financial investment in the collection of data. Geisinger has electronic health record (EHR) data and millions of biomedical images encompassing patient visits across health and disease that have been collected in EPIC® since 1996, with a stable patient population that uses primary and specialty care services. Furthermore, Geisinger has the MyCode Community Health Initiative, a biorepository with a growing collection of genome-wide array data and whole-exome sequencing data that will eventually surpass 200,000 individuals [22]. Thus, there is a unique opportunity to use the existing imaging, clinical, and genetic data from Geisinger for new discoveries on the impact of obesity and the identification of risk factors that can be used as biomarkers in translational medicine. Through the application of advanced image processing and computer vision methods that scale up the use of thousands of images, many challenges to the usage of existing EHR and imaging data for the purposes of research can be surpassed.

As a proof-of-principle study, we used 2,545 distinct CT images from the EHR of Geisinger and used a “greedy snake” segmentation algorithm to automatically measure individual levels of VAT, SAT, and total adipose tissue (TAT), as well as adipose ratio levels. Using ICD-9 codes to define cases and controls for diagnoses and 37 clinical laboratory measures, we characterized associations between these CT-derived adipose measures and a wide range of obesity-related diagnoses as well as quantitative traits. Subsequently, we also performed sex-stratified analyses to identify sex-specific associations with clinical diagnoses and obesity-related traits. Finally, we performed a genome-wide association study (GWAS) between the adipose measures and ∼600,000 common genetic variants and identified suggestive genetic associations.

## 2. Methods

### 2.1. Study Samples

The EHR and genetic data of this study for 2,545 individuals came from Geisinger and the MyCode Community Health Initiative of Geisinger. The genetic data were genotyped as a part of the DiscovEHR collaboration between Geisinger and Regeneron Genetics. Given that most patients within Geisinger are of European American (EA) ancestry (97%), only EA subjects were included in this study (Table 1).

### 2.2. Image Data Collection

Adipose tissue measurements were obtained using an image segmentation technique called the “greedy snake” algorithm that analyzes delineated areas of TAT, VAT, and SAT from preexisting abdominal CT scans within Geisinger's EHR. Data analyses were conducted for adipose measures extracted from the CT slice showing the largest area of total adipose. Also, these same measures from the slice with the largest waist circumference within abdominal CT scans were evaluated.

Our previous work [23] describes the algorithm in great detail; here, we just briefly review the main steps. We formulated automatic fat quantification as an unsupervised contour minimization problem. The proposed algorithm comprised four major parts: (1) data preprocessing, (2) outer body contour estimation, (3) abdominal contour estimation, and (4) adipose quantification. Data preprocessing was done using standard image processing operations. Given an abdominal CT image, the first step was to separate the body from the whole image. This was done with a simple thresholding, taking into account the Hounsfield range of regions of interest. Next, a morphological opening operation was used to remove material artifacts like tables and trays. After segmenting the body from the image, the Moore-neighbor tracing algorithm was used to estimate the outer body contour. In computer vision, active contours are widely used over edge detection algorithms to locate the contour of an object, and they impose energy minimization of properties like smoothness and continuity to make the segmentation robust to noises and edge discontinuities.

The “greedy snake” algorithm differs from the original “active contour” algorithm by computing the movement of each contour point in a discrete manner. The outer body contour is provided as an initial contour to the “greedy snake” algorithm. At each iteration, the algorithm then makes a greedy choice and moves contour points to the position of minimum energy, a linear combination of image energy, elasticity energy, and the curvature energy of the image and the contour points, respectively. In this way, we could identify the body contour and the abdominal cavity contour.

In the last step, we quantified various fat tissues using the body contour and the abdominal cavity contour, respectively. Any pixel within the Hounsfield unit range (−190, −30) was identified as adipose. The TAT region was calculated by the area inside the outer body contour. The VAT region was determined by the area inside the abdominal cavity contour, and finally, the SAT region was calculated by the area between the body and abdominal cavity contours. We also calculated visceral-to-subcutaneous fat ratios (VSRs) and visceral-to-total fat ratios (VTRs) using VAT and SAT. This algorithm identified TAT, VAT, and SAT segmentations with 0.885%, 3.55%, and 3.26% average error, respectively, as compared to a manual segmentation [23].

### 2.3. Evaluation of Measures

Following the extraction of adipose tissue measurements from CT images, each measure type was evaluated for normality of data distribution and for identification of outliers. Adipose tissue measurements showing nonnormal distributions were Box-Cox transformed. Evaluation of all measures was done in R v3.4.0 [24]. Summary statistics of adipose measurements are given in Supplementary Table 1.

### 2.4. ICD-9-Based Diagnoses

International Classification of Disease (ICD-9) diagnosis codes of the Geisinger EHR were used to define the case-control status for diagnoses. Cases were defined as individuals with three or more visits for a specific ICD-9 code at the 5-digit level (e.g., 250.60), whereas controls were defined as individuals having zero visits for the same code. Individuals were excluded from analysis for a given ICD-9 code if they had one to two visits for that specific ICD-9 code. We required at least 50 or more case subjects for the diagnosis to be included in our association testing. Based on our criteria for inclusion/exclusion, we used 1,233 ICD-9 codes for association testing, and those listed in Supplementary Table 2 are the 263 phenotypes where the regression models converged.

### 2.5. Clinical Lab Measures

We had 37 different blood- and serum plasma-derived median/mean clinical laboratory measures from the Geisinger EHR. All clinical laboratory measures were evaluated for normality and outliers, where values greater than 2.5 standard deviation were removed. Log transformations were selectively applied after evaluating clinical laboratory data for normality. Supplementary Table 3 shows the summary statistics of all laboratory measures and additional summary statistics of our dataset.

### 2.6. Genotyping and Quality Control

Genotyping of Geisinger MyCode® participants was done using the Illumina HumanOmniExpress-12 v1.0 array through the DiscovEHR collaboration. Genotype quality control (QC) was performed prior to any association testing, using R 3.4.0 and PLINK [25] for the entire genetic dataset of MyCode (∼38,000 individuals at the time of this study). We filtered single-nucleotide polymorphisms (SNPs) for sample call rates (99%), genotyping (99%), and a minor allele frequency threshold of 1%. Additionally, highly related samples were then removed on the basis of their identity by descent kinship coefficient estimates (pi-hat > 0.125). Principal components were calculated using EIGENSOFT to confirm the EA ancestry status of individuals who also had imaging data. Following QC and filtering for samples that had both genotyping and adipose imaging data, we had 629,675 SNPs and 2,545 EA samples.

### 2.7. Associations between CT-Derived Adipose Measures and Diagnoses and Clinical Laboratory Measures

Associations between the CT-derived adipose measures and ICD-9-derived diagnoses or clinical laboratory measures were calculated using logistic regression and linear regression, respectively, using the software PLATO [26]. In both types of analyses, the responses were adjusted for age and sex. We also performed the analyses adjusted for age, sex, and type 2 diabetes status to determine if there were any appreciable differences in associations with those covariates. In addition to determining strength of associations between adipose measures and ICD-9 codes, odds ratios and 95% confidence intervals were also calculated in PLATO. Analyses were then repeated, stratified by sex, where age was used as a covariate.

### 2.8. Associations between CT-Derived Adipose Measures and Diagnoses and Clinical Laboratory Measures for WC and TAT

We evaluated ICD-9 diagnosis associations from the optical slice with the greatest TAT, as well as ICD-9 diagnosis associations from the slice with the greatest WC. We did see a difference in the significance of associations when using the TAT measures compared to WC measures, for visceral adipose, but not subcutaneous adipose. All VAT and SAT association results presented within this manuscript are for associations from the imaging slices with greatest TAT.

### 2.9. Bonferroni Correction and False Discovery Rate for Association Tests between Adipose Measures and EHR Data

A Bonferroni correction of 2.01 × 10^−5^ (i.e., (1 × 10^−2^)/(1,233 × 4)) (the denominator corresponds to 1,233 ICD-9 codes and 4 phenotypes) was used as a significance threshold for association tests using ICD-9 code diagnoses and 7.14 × 10^−5^ (i.e., (1 × 10^−2^)/(35 × 4)) (the denominator corresponds to 35 clinical lab measures and 4 phenotypes) was used in the association tests using clinical laboratory measures. In addition to the Bonferroni threshold, a false discovery rate (FDR) of 1% was determined. This translated to a level of significance of 2.09 × 10^−4^ for analyses using logistic regression and 2.57 × 10^−3^ for analyses using linear regression. A 1% FDR for the sex-stratified dataset translated into a level of significance of 1.58 × 10^−4^ and 2.56 × 10^−3^ for females and 1.38 × 10^−4^ and 3.09 × 10^−3^ for males for logistic regression and linear regression, respectively.

### 2.10. Genetic Associations

Genetic associations between 629,675 SNPs obtained after QC and adipose tissue measures (VAT, SAT, VSR, and VTR) were calculated in PLATO using age and sex as covariates. Following the GWAS, SNPs were annotated using the NHGRI-EBI GWAS catalog [27] and GRASP [28] to determine if any associations within this study were replicated in previous studies, using a reported *p* value significance of at least 1 × 10^−4^ from the GWAS catalog and GRASP.

## 3. Results

Summary statistics of all phenotypes can be found in Table 1. Our study had a total of 2,545 subjects, including 1,238 men and 1,307 women, with a mean age of 54 ± 17 (mean ± standard deviation) years.

### 3.1. Associations between Adipose Tissue Measures and Clinical Diagnosis Codes

**Figure 1:**
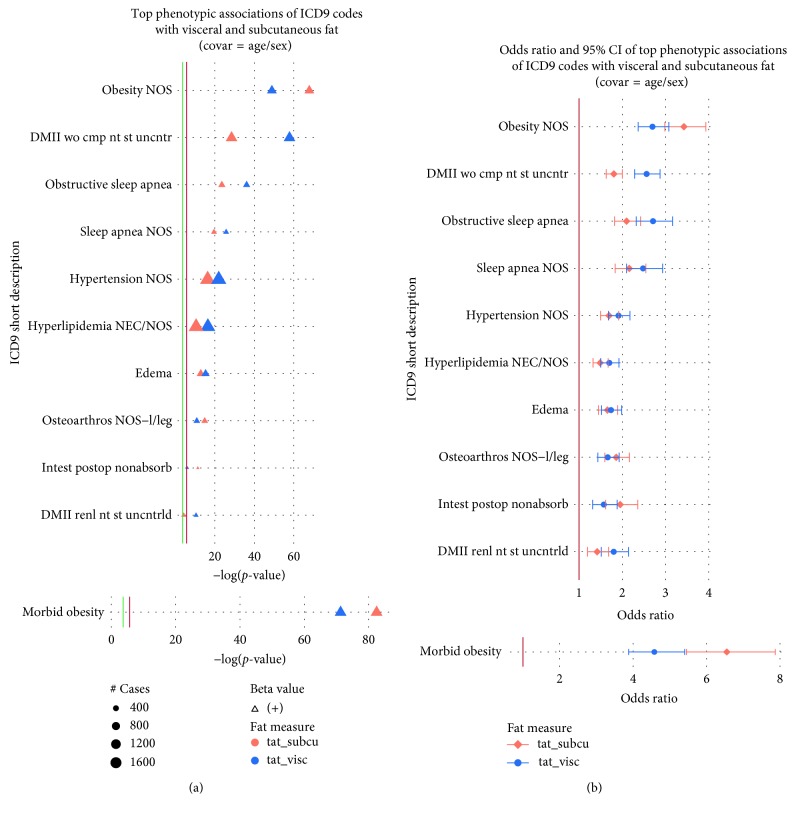
(a) The plot shows −log(*p* values) for the top phenotypic associations between ICD-9-based diagnoses on the *y*-axis for VAT and SAT after controlling for age and sex. Point size indicates the number of cases (approximately 400, 800, 1200, and 1600). The direction of the point, upwards or downwards, represents the direction of the corresponding beta estimate (positive or negative) (b) Odds ratios and 95% confidence intervals (CIs) of top phenotypic associations of ICD-9 codes with VAT and SAT after controlling for age and sex. In both panels, SAT is represented in orange and VAT in blue. The red line corresponds to the Bonferroni threshold, whereas the green line corresponds to the 1% FDR threshold. Refer to the full descriptions of the ICD-9 code abbreviations in Supplementary Table 2.

The results of all associations between adipose tissue and clinical diagnosis codes are presented in Supplementary Figure 1, and Supplementary Table 4 shows association results, with *p* values <1 × 10^−5^, of SAT and VAT with ICD-9 codes for sex-combined analyses. The most significant associations across all the diagnoses were between our CT-derived adipose tissue measures (VAT and SAT) and obesity-related ICD-9 diagnostic codes.  Figure 1 shows both the *p* values plotted with −log 10(*p* value) as well as the odds ratios (ORs) and confidence intervals (CIs) for the results passing our 1% FDR cutoff (refer to the full descriptions of the ICD-9 code abbreviations in Supplementary Table 2). The associations with morbid obesity were more significant by 10–20 orders of magnitude; therefore, the results were moved to a separate plot to maintain reasonable axes for the rest of the results. The results show the majority of the associations were positive and thus were associated with increased risk.  Figure 1(a) shows SAT had a stronger association with both morbid obesity (*p* = 3.29 × 10^−83^; 95% CI = [5.45, 7.87]) and “obesity unspecified” (obesity NOS) (*p* = 1.41 × 10^−68^; 95% CI = [2.98, 3.93]). However, out of the top results, VAT had a stronger association than SAT with other obesity-related ICD-9 codes such as “diabetes mellitus without mention of complication, type II, or unspecified type, not stated as controlled” (DMII wo cmp nt st uncrntr) (VAT: *p* = 1.49 × 10^−58^; 95% CI = [2.29, 2.87], over SAT: *p* = 3.23 × 10^−29^; 95% CI = [1.63, 2.00]) (Figures 1(a) and 1(b)). While the −log(*p* values) and OR show trends of stronger associations with clinical diagnoses and VAT, the CIs for many of these same associations overlap between SAT and VAT.

**Figure 2:**
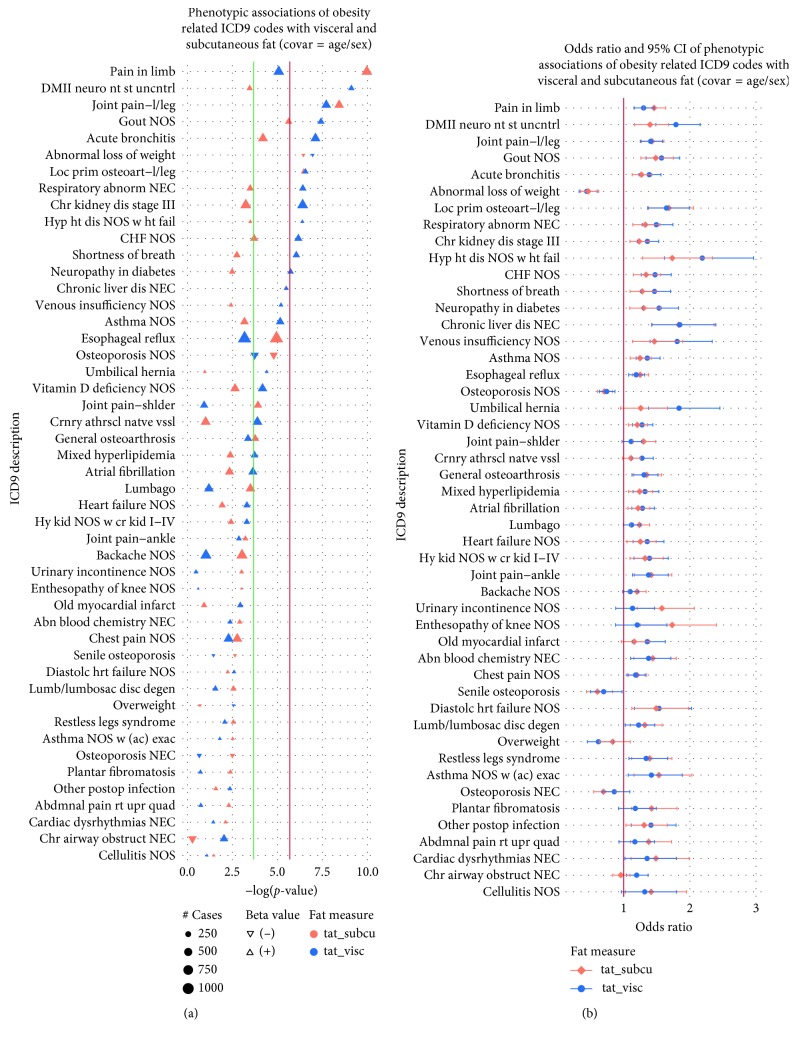
(a) The plot shows −log(*p* values) for phenotypic associations of obesity-related ICD-9-based diagnoses with VAT and SAT on the *y*-axis after controlling for sex and age. Point size refers to the number of cases (250, 500, 750, and 1000). The direction of the point, upwards or downwards, represents the direction of the corresponding beta estimate (positive or negative) (b) Odds ratios and 95% CIs of phenotypic associations of obesity-related ICD-9 codes with VAT and SAT after controlling for sex and age. In both panels, SAT is represented in orange and VAT in blue. The red line corresponds to the Bonferroni threshold, whereas the green line corresponds to the 1% FDR threshold.

In Figure 2, we filtered the total associations for phenotypes that are related to obesity (e.g., comorbidities but not specifically obesity diagnoses). Again, the majority of associations showed an increased risk for obesity-related comorbidities. The only notable divergence from this was a decreased risk of osteoporosis, and there are conflicting reports regarding the relationship between obesity and bone mass [29]. As with the results of Figure 1, VAT generally showed stronger associations with obesity-related comorbidities (Figure 2(a)) as well as clinical diagnoses (Figure 2(b)); however, their respective CIs showed an overlap between SAT and VAT (Figure 2(b)).

### 3.2. Associations between Adipose Tissue Measures and Clinical Laboratory Measures

**Figure 3:**
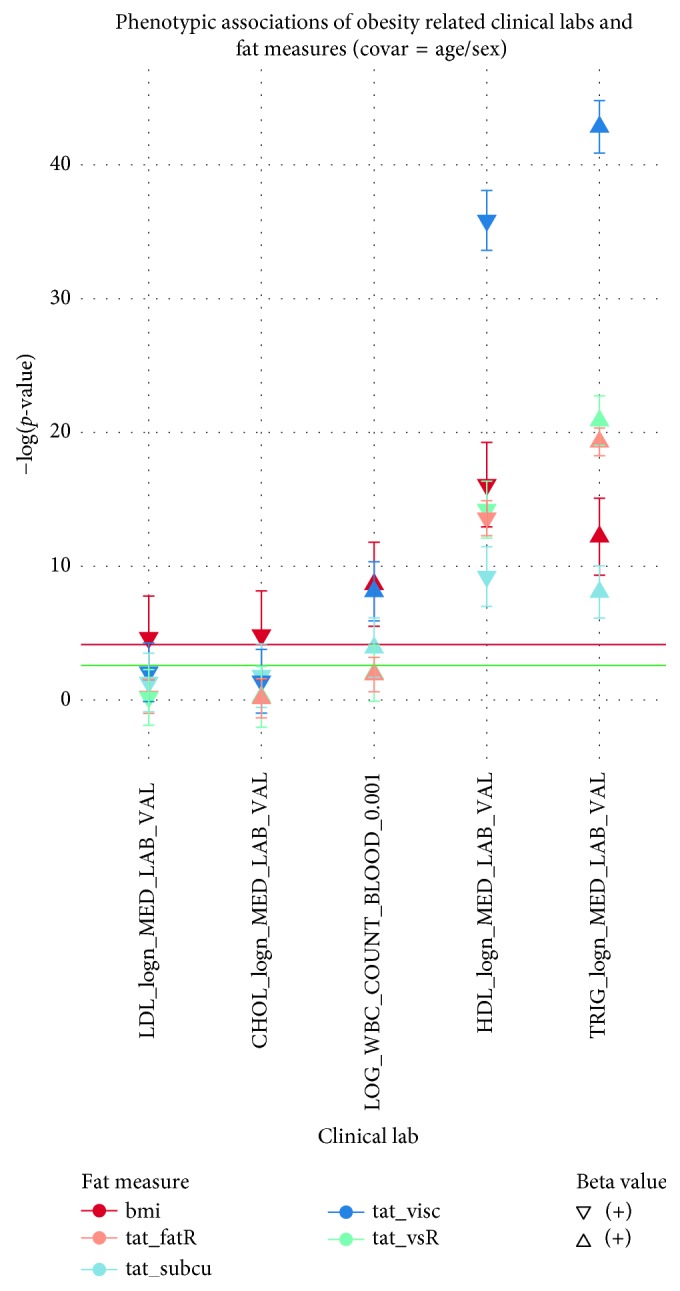
This plot shows −log(*p* values) for adipose measures (*y*-axis: BMI in red, fat ratio in yellow, SAT in light blue, VAT in blue, and VSR in green) associated with obesity-related clinical laboratory measures (*x*-axis: natural log-transformed low-density lipoprotein (LDL), cholesterol (CHOL) levels, high-density lipoprotein (HDL), triglycerides (TRIG), and log-transformed white blood cell (WBC) counts after controlling for age and sex). The direction of the point, upwards or downwards, represents the direction of the corresponding beta estimate (positive or negative). The red line corresponds to the Bonferroni threshold, whereas the green line corresponds to the 1% FDR threshold.

Clinical lab measures obtained from outpatient clinics provided an opportunity to determine the relationship between adiposity and measures such as high-density lipoprotein (HDL) levels, low-density lipoprotein (LDL) levels, cholesterol (CHOL) levels, triglyceride (TRIG) levels, and white blood cell (WBC) counts. Supplementary Table 5 presents results of sex-combined associations between SAT/VAT and clinical lab measures with *p* values <1 × 10^−1^, and Supplementary Table 6 provides summary statistics for the clinical lab measures. All obesity and adipose measures had similar directions of effect for associations with the clinical lab measures. However, VAT showed the most significant association for lipid levels, having the strongest association with both HDL (*p* = 1.42 × 10^−36^; standard error (SE) = 5.80 × 10^−3^) and TRIG (*p* = 1.44 × 10^−43^; SE = 1.08 × 10^−2^) (Figure 3). Additionally, the adipose measures showed a negative direction of association with HDL (VAT *β* = −0.075) but a positive direction of association with TRIG (VAT *β* = 0.153). Interestingly, VAT also showed a significantly higher positive association with WBC (*p* = 7.37 × 10^−9^; SE = 6.15 × 10^−3^) compared to SAT (*p* = 1.21 × 10^−4^; SE = 6.04 × 10^−3^), although there was little difference from BMI (*p* = 2.21 × 10^−9^; SE = 7.22 × 10^−4^) (Figure 3).

### 3.3. Sex-Stratified Analyses

To identify different trends of adipose deposition associated with health outcomes for men and women, we performed the same associations as above, sex stratified by both clinical diagnosis codes and lab measures. Summary statistics of sex-stratified clinical labs can be found in Supplementary Table 7. The results described here are limited to ICD-9 codes that were found to be associated in both sexes (some diagnoses are specific to women only and vice versa and thus would only be evaluated in one group or the other). Although the number of women and the number of men were very closely balanced, the strength of association for several different health outcomes was greater for women.

**Figure 4:**
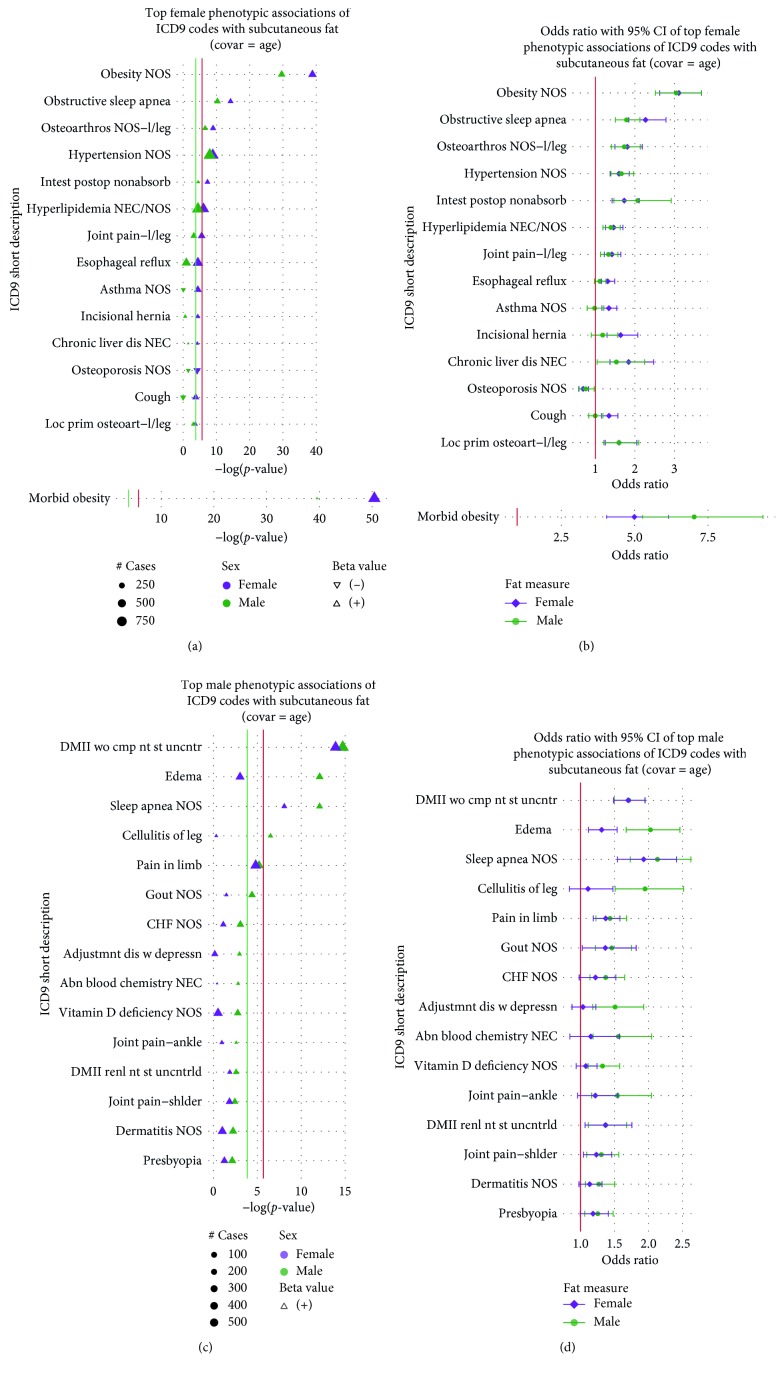
The plot shows −log(*p* values) for top sex-stratified phenotypic associations of ICD-9-based diagnoses with SAT on the *y*-axis after controlling for age. (a) Top female phenotypic associations of ICD-9 codes with SAT. Point size refers to the number of cases (250, 500, and 750). (b) Odds ratios and 95% CIs of top female phenotypic associations of ICD-9 codes with SAT after controlling for age. (c) Top male phenotypic associations of ICD-9 codes with SAT. Point size refers to the number of cases (100, 200, 300, 400, and 500). (d) Odds ratios and 95% CIs of top male phenotypic associations of ICD-9 codes with SAT after controlling for age. In both panels, females are represented in purple and males in green. The direction of the point, upwards or downwards, represents the direction of the corresponding beta estimate (positive or negative). The red line corresponds to the Bonferroni threshold, whereas the green line corresponds to the 1% FDR threshold.

Most notably, compared to males, SAT in females showed stronger associations with morbid obesity (*p* = 3.85 × 10^−51^; 95% CI = [4.04, 6.15]), unspecified obesity (obesity NOS) (*p* = 1.32 × 10^−39^; 95% CI = [2.63, 3.69]), and “obstructive sleep apnea” (*p* = 5.42 × 10^−15^; 95% CI = [1.85, 2.79]) (Figure 4(a)), although the ORs again had overlapping CIs (Figure 4(b)). On the contrary, SAT in males, compared to females, showed a higher association with “edema” (*p* = 7.87 × 10^−13^; 95% CI = [1.67, 2.46]) and “cellulitis of the leg” (*p* = 3.05 × 10^−7^; 95% CI = [1.51, 2.52]) (Figures 4(c) and 4(d)). Beyond the top associations, neither sex showed additional associations for SAT that met at least the 1% FDR threshold (Supplementary Figures 2(a) and 2(c)).

**Figure 5:**
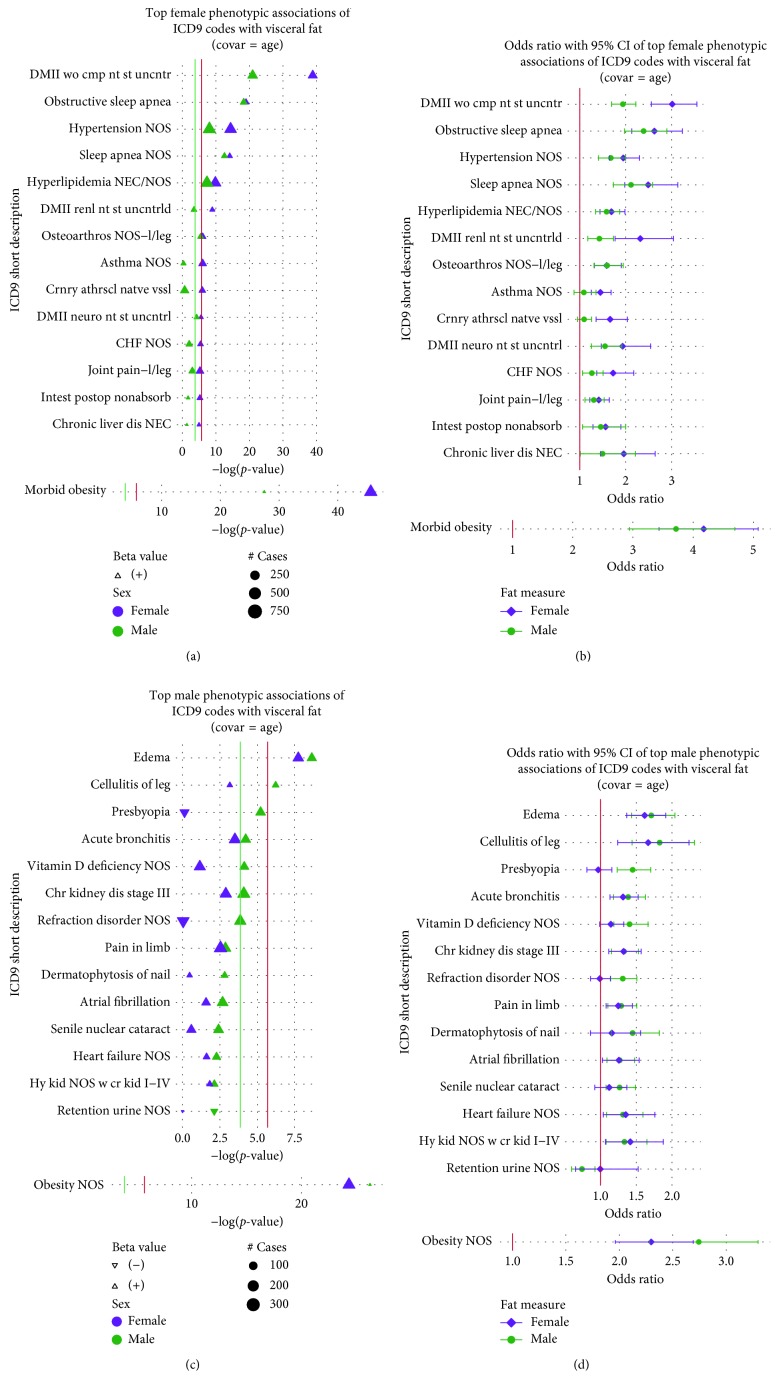
The plot shows −log(*p* values) for top sex-stratified phenotypic associations of ICD-9-based diagnoses with VAT on the *y*-axis after controlling for age. (a) Top female phenotypic associations of ICD-9 codes with VAT. Point size refers to the number of cases (250, 500, and 750). (b) Odds ratios and 95% CIs of top female phenotypic associations of ICD-9 codes with VAT after controlling for age. (c) Top male phenotypic associations of ICD-9 codes with VAT. Point size refers to the number of cases (100, 200, and 300). (d) Odds ratios and 95% CIs of top male phenotypic associations of ICD-9 codes with VAT after controlling for age. In all panels, females are represented in purple and males in green. The direction of the point, upwards or downwards, represents the direction of the corresponding beta estimate (positive or negative). The red line corresponds to the Bonferroni threshold, whereas the green line corresponds to the 1% FDR threshold.

The top associations between ICD-9 codes and VAT also showed sex specificity but differed from associations found in sex-combined analyses. Females showed strong evidence of VAT associations with “diabetes mellitus without the mention of complication, type II, or unspecified type, not stated as controlled” (*p* = 1.34 × 10^−39^; 95% CI = [2.55, 3.55]), “diabetes with renal manifestations, type II, or unspecified type, not stated as uncontrolled” (DMII renl nt st uncntrld). (*p* = 1.18 × 10^−9^; 95% CI = [1.77, 3.04]), and “coronary atherosclerosis” (Crnry athrscl natve vssl) (*p* = 1.11 × 10^−6^; 95% CI = [1.35, 2.04]), with all relationships having significantly different ORs than males (Figures 5(a) and 5(b)). Outside of these top results, VAT associations for five ICD-9 codes (“shortness of breath,” “gout” (Gout NOS), “osteoporosis” (Osteoporosis NOS), “neuropathy in diabetes,” and “osteoarthritis of lower leg” (Loc prim osteoart-l/leg)) reached 1% FDR-level significance in females (1.58 × 10^−4^) compared to none in males (Supplementary Figures 3(a) and 3(c)). Even beyond the FDR significant results, females showed a stronger and greater number of relationships between VAT and ICD-9 codes compared to males, although ORs did not significantly differ between sexes (Supplementary Figures 3(B) and 3(D)). Finally, like with SAT, males had a stronger VAT association with “edema” (*p* = 2.27 × 10^−9^; 95% CI = [1.43, 2.04]) and “cellulitis of the leg” (*p* = 5.94 × 10^−7^; 95% CI = [1.44, 2.31]); however, there were overlapping CIs for the ORs (Figures 5 and 5). A full summary of sex-stratified association testing for ICD-9 codes, with *p* values < 1 × 10^−2^, can be found in Supplementary Table 8.

**Figure 6:**
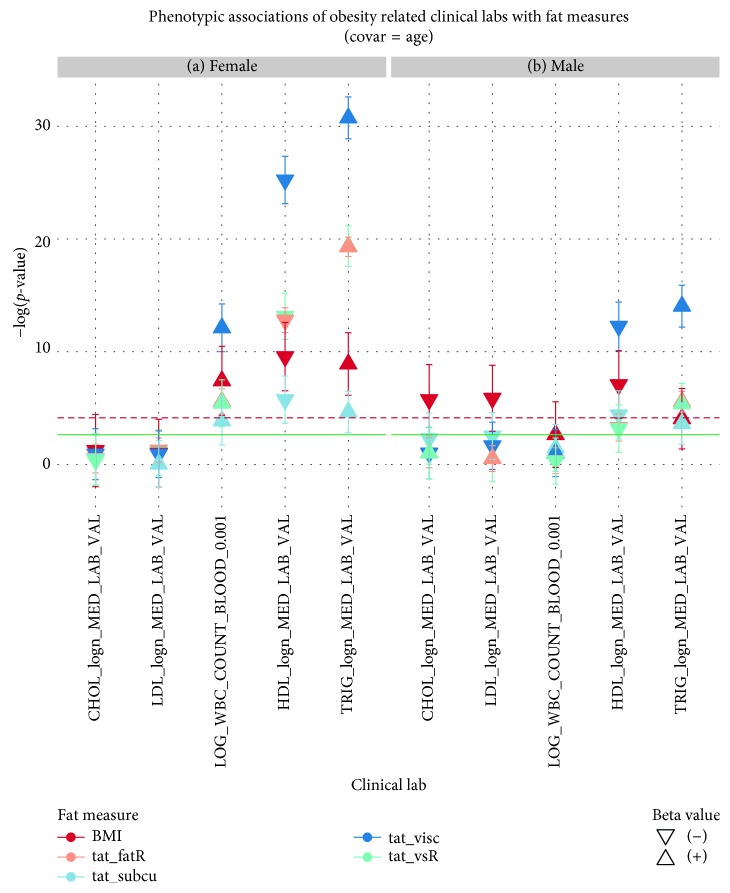
(a) This plot shows −log(*p* values) for adipose measures for females (*y*-axis: BMI in red, fat ratio in yellow, SAT in light blue, VAT in blue, and VSR in green) associated with obesity-related clinical laboratory measures (*x*-axis: natural log-transformed LDL, CHOL, HDL, TRIG, and log-transformed WBC) after controlling for age. (b) This plot shows −log(*p* values) for adipose measures for males (*y*-axis: BMI in red, fat ratio in yellow, SAT in light blue, VAT in blue, and VSR in green) associated with obesity-related clinical laboratory measures (*x*-axis: natural log-transformed LDL, CHOL, HDL, TRIG, and log-transformed WBC) after controlling for age. The direction of the point (facing upwards or downwards) in both panels represents the direction of the corresponding beta estimate (positive or negative). The red line corresponds to the Bonferroni threshold, whereas the green line corresponds to the 1% FDR threshold.

Similar to the sex-combined analyses, key clinical laboratory measures were tested for their associations with adipose tissue measures. In females, VAT showed the strongest association with WBC count (*p* = 7.49 × 10^−13^; SE = 7.58 × 10^−3^), HDL (*p* = 5.94 × 10^−26^; SE = 7.95 × 10^−3^), and TRIG (*p* = 1.84 × 10^−31^; SE = 1.39 × 10^−2^), whereas in males, VAT showed the strongest associations with HDL (*p* = 5.50 × 10^−13^; SE = 7.04 × 10^−3^) and TRIG (*p* = 9.03 × 10^−15^; SE = 1.39 × 10^−2^) (Figures 6 and 6). The direction of effect was similar to that of the sex-combined analyses. While both sexes showed a strong association between VAT and HDL as well as VAT and TRIG, females showed evidence of a stronger association with key obesity-related laboratory measures than males. This observation lends further support to the notion that visceral fat levels may impact obesity disease outcomes to a greater extent in females compared to males. A summary of the full results of sex-stratified clinical lab association testing can be found in Supplementary Table 9.

### 3.4. Genome-Wide Association Studies of Adipose Tissue

Manhattan plots for sex-combined analyses are presented in Supplementary Figures 4–7, and a summary of the top results can be found in Supplementary Table 10. Among all genome-wide associations tests, only one SNP (rs10743966) reached genome-wide significance (*p* = 2.97 × 10^−8^ for SAT; Supplementary Figure 5) and has previously been shown to be associated with WHR [30] and CVD [31]. Although no other SNPs reached statistical significance, GRASP annotations of the top results of SAT and VAT reveal previously established associations with obesity-related phenotypes. Additionally, SNPs associated with calculated adipose ratios (VSR and VTR) were also previously associated with obesity-related phenotypes. More specifically, the top SNP associated with VAT (rs933186; *p* = 1.29 × 10^−7^) was previously shown to be related to HDL cholesterol [32, 33], while the top SNP associated with VSR (rs12950848; *p* = 6.45 × 10^−7^) was previously shown to be related to lipid levels [32, 33] and total cholesterol [32]. Lastly, although VTR's top association, rs7699631 (*p* = 1.99 × 10^−6^), has no known associations with obesity-related traits, it was found to have a strong association with rs12950848 (*p* = 2.61 × 10^−6^) which was earlier shown to be associated with LDL cholesterol [32].

## 4. Discussion

The level of adipose tissue individuals carry has been previously shown to be an important risk factor for several obesity-related diseases. While BMI is typically used as a measurement of adipose levels, the location of adiposity is not reflected in this measure. Waist circumference is another measure that provides a reflection of the location of adiposity, in addition to the amount, as there is a known relationship between improved health outcomes for “pear-shaped” adiposity compared to “apple-shaped,” or more central, adiposity. Although BMI and waist-to-hip ratio have been shown to be predictors of CVD and mortality, they are not effective in distinguishing subcutaneous adipose from visceral adipose. Visceral adipose, the fat closest to the internal organs and more centrally deposited, is believed to have the most important impact on metabolic disorders, and increased visceral adipose has been linked to hypertension, atherosclerosis, and diabetes [34–36]. Individuals with high BMI can have a high level of physical fitness and a lower risk of mortality as compared to individuals with lower BMI but a different distribution of adiposity [34]. Women generally have a higher amount of body fat than men; however, women tend to carry excess adipose in their hips and thighs (gluteal-femoral region) [37, 38], whereas men carry it around their abdomen.

In this paper, we present a proof-of-principle study where we used imaging data from CT scans collected within a health system and then used a high-throughput automated approach to obtain measures of subcutaneous and visceral adipose tissue across individuals. An added advantage to this approach is that the imaging data can be coupled with the health data recorded on patients, including patients' diagnoses as well as their respective clinical laboratory measures. With this new approach, it is important to show we recapitulate known trends and relationships between adipose types, diagnoses, and clinical lab measures as a proof of principle in addition to potentially new discoveries.

Our results show that obesity-related ICD-9 diagnostic codes have strong associations with VAT and SAT. In particular, morbid obesity was more strongly associated with SAT as compared to VAT. These results do reinforce that subcutaneous adipose levels provide an accurate reflection of the degree of obesity for an individual. Importantly, however, in line with the significant impact of visceral adipose on obesity-related conditions and comorbidities, our study shows the strength of the majority of associations is higher for VAT compared to SAT. The direction of effect of the majority of associations with obesity-related outcomes was for increased risk of these outcomes with increases in VAT.

Intriguingly, even with balanced numbers of men and women and no major difference in power due to sample size, we saw the associations between SAT/VAT and obesity or obesity-related comorbidities to be consistently more significant for women than men. This will be an important area to research in a larger study with more CT-derived adipose measures to further determine the impact of these adipose levels when stratifying by sex. If these results replicate in a larger sample size, this could point to the increased importance on health outcomes of centralized adiposity for women versus men.

In the context of clinical lab measures, the negative direction of association for HDL, but a positive direction of association for triglycerides, particularly implies that the increase in VAT is associated with increased levels of triglycerides but decreased levels of HDL. HDL is considered a beneficial circulating lipid, while triglycerides are not, so this association points to the critical importance of VAT's effect on obesity-related risks compared to BMI alone. Also, the significantly higher positive association of WBC with VAT compared to SAT may be related to increasing inflammation with obesity [39, 40]. Our clinical lab associations also suggest that the presence of a greater volume of visceral tissue in females may impact the outcomes of more obesity-related diseases than in males, as we again saw a more significant magnitude of association with these clinical lab measures in women compared to men. For this study, we used the median clinical lab measure each individual had across the lipid measures they had recorded in the EHR. In the future work, we will explore both the relationships of longitudinal lipid and blood cell count measures with visceral and subcutaneous adipose measures, characterizing the impact of medications such as lipid-lowering drugs on results when taken into account.

Notably, for the majority of the associations of this study, except for the most statistically significant ones, there were overlapping confidence intervals for the association results of VAT and SAT. Thus, while we saw trends of the magnitude of association being higher for VAT compared to SAT for many diagnoses, the overlapping confidence intervals do not support a truly statistically significant difference. Power is always a consideration in association testing, and while ICD-9 codes have shown successful association testing in other studies [41], they can increase noise and decrease power. Thus, to strengthen these initial findings further, our future direction will be to deploy this strategy across thousands of CT images, thereby increasing our power to detect associations. We also had few statistically significant genetic associations and again that could be attributed to power for this small proof-of-principle study, and our future plan is to repeat these associations with larger sample sizes.

Overall, the results of this study show the utility of repurposing existing imaging data for the study of the impact of visceral adipose levels on health outcomes. These images, while collected in large numbers, are an incredible untapped resource for a wide range of research projects. This project also shows the importance of using automated phenotype extraction from imaging datasets. Manual segmentation of images would have been considerably more time-consuming and will not scale up to the future work with measures obtained from additional CT scans. With the ever-increasing number of medical images available within electronic health record systems, using these images in a high-throughput manner is a powerful resource for research. Through this approach, we can obtain large sample sizes of understudied quantitative measures and subphenotypes beyond clinical lab measures, health screening measures such as BMI, and diagnoses. These measures obtained from images may better reflect the complexity of diseases and comorbidities, opening the door to new discoveries of the impact of these measures on health and the relationship to the genetic architecture.

## Figures and Tables

**Table 1:** Demographics and descriptive information of the cohort.

Covariate	Value	Count or (min, median, max)
Sex	Female	1307
	Male	1238
Diabetes	0	1796
	1	749
Age	<18	3
	19–40	402
	41–60	919
	61–80	1046
	80+	175
Weight (kg)	—	(36.27, 88.45, 188.69)
Height (m)	—	(1.09, 1.69, 2.52)
BMI (kg/m^2^)	—	(12.56, 30.72, 68.48)

## Data Availability

The electronic health record and genetic data used to support the findings of this study have not been made available because of protecting patient privacy.

## Supplementary Materials

Supplementary Materials Supplementary Figure 1: the plot shows −log(*p*-values) for all phenotypic associations between ICD-9-based diagnoses on the *y*-axis for both VAT and SAT after controlling for age and sex. Point size is indicative of the number of cases (500, 1000, and 1500), and the direction of the point, upwards or downwards, represents the direction of the beta estimate (positive or negative). Supplementary Figure 2: the plot shows −log(*p*-values) for sex-stratified phenotypic associations, outside of the top results, of ICD-9-based diagnoses (*y*-axis) associated with SAT after controlling for age. (A) Results of female phenotypic associations of ICD-9 codes with SAT, after controlling for age, which show a stronger relationship for females compared to males. (B) Results of odds ratios and 95% CIs of female phenotypic associations of ICD-9 codes with SAT after controlling for age. (C) Results of male phenotypic associations of ICD-9 codes with SAT, after controlling for age, which show a stronger relationship for males compared to females. (D) Results of odds ratios and 95% CIs of male phenotypic associations of ICD-9 codes with SAT after controlling for age. Supplementary Figure 3: the plot shows −log(*p*-values) for sex-stratified phenotypic associations, outside of the top results, of ICD-9-based diagnoses (*y*-axis) associated with VAT after controlling for age. (A) Results of female phenotypic associations of ICD-9 codes with VAT, after controlling for age, which show a stronger relationship for females compared to males. (B) Results of odds ratios and 95% CIs of female phenotypic associations of ICD-9 codes with VAT after controlling for age. (C) Results of male phenotypic associations of ICD-9 codes with VAT, after controlling for age, which show a stronger relationship for males compared to females. (D) Results of odds ratios and 95% CIs of male phenotypic associations of ICD-9 codes with VAT after controlling for age. Supplementary Figure 4: Manhattan plots showing results for sex-combined analyses of genotypic associations of common variant SNPs with all adipose measures after controlling for age and sex. (A) Manhattan plot for genome-wide association tests for VAT. (B) Manhattan plot for genome-wide association tests for SAT. (C) Manhattan plot for genome-wide association tests for VSR. (D) Manhattan plot for genome-wide association tests for VTR. Blue line denotes a *p*-value of 1 × 10^−5^. Red line indicates a GWAS significance of 5.0 × 10^−8^. Supplementary Table 1: summary statistics of adipose measures. Supplementary Table 2: ICD-9 summary and descriptions. Supplementary Table 3: summary statistics of all EHR data. Supplementary Table 4: cross-phenotype associations of ICD-9 diagnostic codes. Supplementary Table 5: cross-phenotype associations of clinical lab measures. Supplementary Table 6: summary statistics of clinical laboratory measures. Supplementary Table 7: summary statistics of clinical laboratory measures stratified by sex. Supplementary Table 8: sex-stratified association tests for ICD-9 codes. Supplementary Table 9: sex-stratified association testing for clinical labs. Supplementary Table 10: top GWAS findings.
