# Supplementary material for: Using Adipose Measures from Health Care Provider-Based Imaging Data for Discovery
Source: J Obes. 2018 Sep 27;2018:3253096. doi: 10.1155/2018/3253096 (PMC6180992; doi:10.1155/2018/3253096)
Supplement: Supplementary Materials — Supplementary Figure 1: the plot shows −log(p-values) for all phenotypic associations between ICD-9-based diagnoses on the y-axis for both VAT and SAT after controlling for age and sex. Point size is indicative of the number of cases (500, 1000, and 1500), and the direction of the point, upwards or downwards, represents the direction of the beta estimate (positive or negative). Supplementary Figure 2: the plot shows −log(p-values) for sex-stratified phenotypic associations, outside of the top results, of ICD-9-based diagnoses (y-axis) associated with SAT after controlling for age. (A) Results of female phenotypic associations of ICD-9 codes with SAT, after controlling for age, which show a stronger relationship for females compared to males. (B) Results of odds ratios and 95% CIs of female phenotypic associations of ICD-9 codes with SAT after controlling for age. (C) Results of male phenotypic associations of ICD-9 codes with SAT, after controlling for age, which show a stronger relationship for males compared to females. (D) Results of odds ratios and 95% CIs of male phenotypic associations of ICD-9 codes with SAT after controlling for age. Supplementary Figure 3: the plot shows −log(p-values) for sex-stratified phenotypic associations, outside of the top results, of ICD-9-based diagnoses (y-axis) associated with VAT after controlling for age. (A) Results of female phenotypic associations of ICD-9 codes with VAT, after controlling for age, which show a stronger relationship for females compared to males. (B) Results of odds ratios and 95% CIs of female phenotypic associations of ICD-9 codes with VAT after controlling for age. (C) Results of male phenotypic associations of ICD-9 codes with VAT, after controlling for age, which show a stronger relationship for males compared to females. (D) Results of odds ratios and 95% CIs of male phenotypic associations of ICD-9 codes with VAT after controlling for age. Supplementary Figure 4: Manhattan plots showing results for s [file 3253096.f1.zip › 3253096.f1/Supplementary Figure 2_JOBE_2418543.pdf]

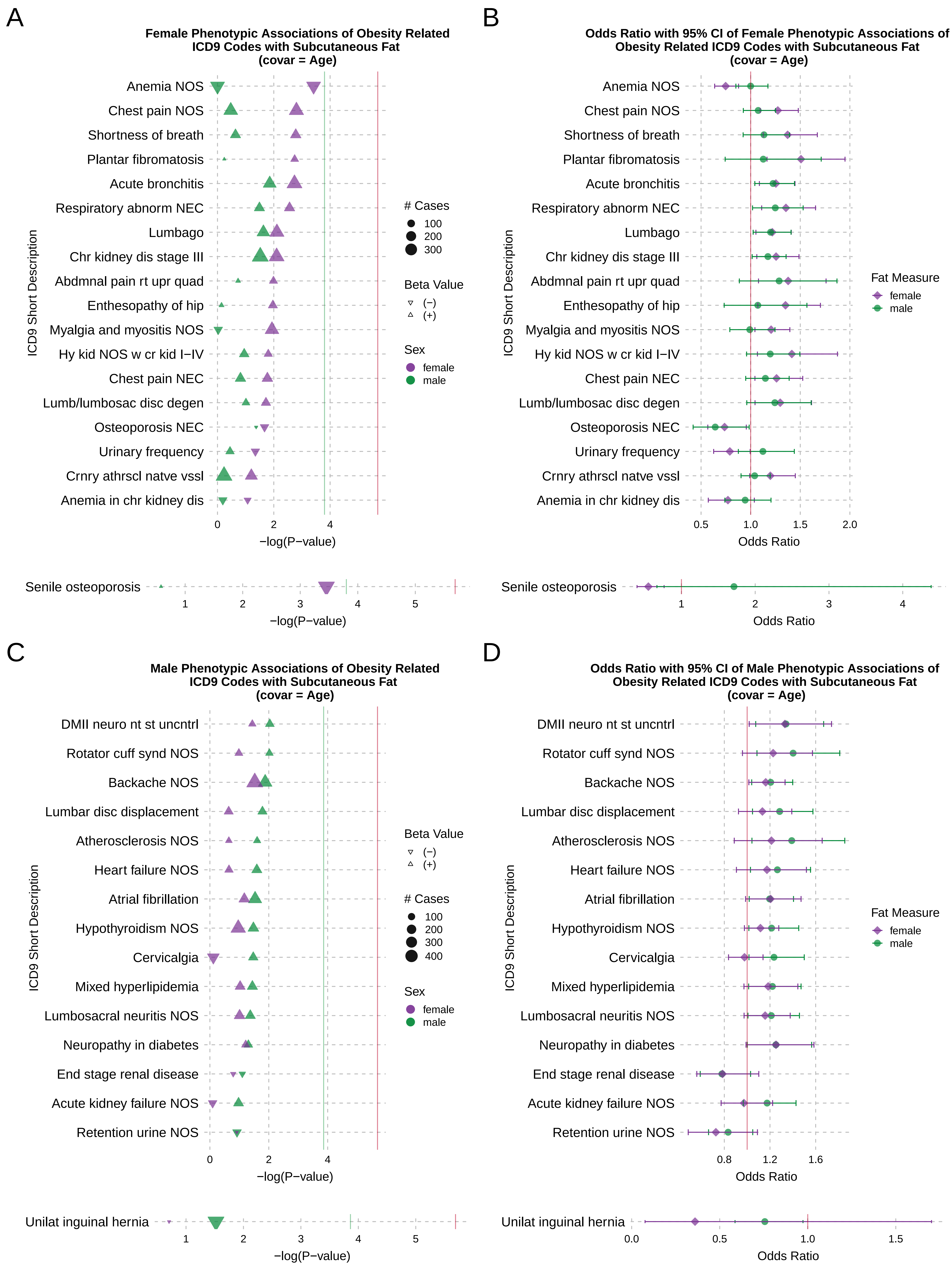
A
Female Phenotypic Associations of Obesity Related ICD9 Codes with Subcutaneous Fat (covar = Age)
ICD9 Short Description
Anemia NOS
Chest pain NOS
Shortness of breath
Plantar fibromatosis
Acute bronchitis
Respiratory abnorm NEC
Lumbago
Chr kidney dis stage III
Abdmnal pain rt upr quad
Enthesopathy of hip
Myalgia and myositis NOS
Hy kid NOS w cr kid I−IV
Chest pain NEC
Lumb/lumbosac disc degen
Osteoporosis NEC
Urinary frequency
Crnry athrscl natve vssl
Anemia in chr kidney dis
−log(P−value)
Cases
100
200
300
Beta Value
(−)
(+)
Sex
female
male
Senile osteoporosis
B
Odds Ratio with 95% CI of Female Phenotypic Associations of Obesity Related ICD9 Codes with Subcutaneous Fat (covar = Age)
Odds Ratio
Fat Measure
female
male
C
Male Phenotypic Associations of Obesity Related ICD9 Codes with Subcutaneous Fat (covar = Age)
DMII neuro nt st uncntrl
Rotator cuff synd NOS
Backache NOS
Lumbar disc displacement
Atherosclerosis NOS
Heart failure NOS
Atrial fibrillation
Hypothyroidism NOS
Cervicalgia
Mixed hyperlipidemia
Lumbosacral neuritis NOS
Neuropathy in diabetes
End stage renal disease
Acute kidney failure NOS
Retention urine NOS
400
Unilat inguinal hernia
D
Odds Ratio with 95% CI of Male Phenotypic Associations of Obesity Related ICD9 Codes with Subcutaneous Fat (covar = Age)
