# Supplementary material for: Using Adipose Measures from Health Care Provider-Based Imaging Data for Discovery
Source: J Obes. 2018 Sep 27;2018:3253096. doi: 10.1155/2018/3253096 (PMC6180992; doi:10.1155/2018/3253096)
Supplement: Supplementary Materials — Supplementary Figure 1: the plot shows −log(p-values) for all phenotypic associations between ICD-9-based diagnoses on the y-axis for both VAT and SAT after controlling for age and sex. Point size is indicative of the number of cases (500, 1000, and 1500), and the direction of the point, upwards or downwards, represents the direction of the beta estimate (positive or negative). Supplementary Figure 2: the plot shows −log(p-values) for sex-stratified phenotypic associations, outside of the top results, of ICD-9-based diagnoses (y-axis) associated with SAT after controlling for age. (A) Results of female phenotypic associations of ICD-9 codes with SAT, after controlling for age, which show a stronger relationship for females compared to males. (B) Results of odds ratios and 95% CIs of female phenotypic associations of ICD-9 codes with SAT after controlling for age. (C) Results of male phenotypic associations of ICD-9 codes with SAT, after controlling for age, which show a stronger relationship for males compared to females. (D) Results of odds ratios and 95% CIs of male phenotypic associations of ICD-9 codes with SAT after controlling for age. Supplementary Figure 3: the plot shows −log(p-values) for sex-stratified phenotypic associations, outside of the top results, of ICD-9-based diagnoses (y-axis) associated with VAT after controlling for age. (A) Results of female phenotypic associations of ICD-9 codes with VAT, after controlling for age, which show a stronger relationship for females compared to males. (B) Results of odds ratios and 95% CIs of female phenotypic associations of ICD-9 codes with VAT after controlling for age. (C) Results of male phenotypic associations of ICD-9 codes with VAT, after controlling for age, which show a stronger relationship for males compared to females. (D) Results of odds ratios and 95% CIs of male phenotypic associations of ICD-9 codes with VAT after controlling for age. Supplementary Figure 4: Manhattan plots showing results for s [file 3253096.f1.zip › 3253096.f1/Supplementary Figure 3_JOBE_2418548.pdf]

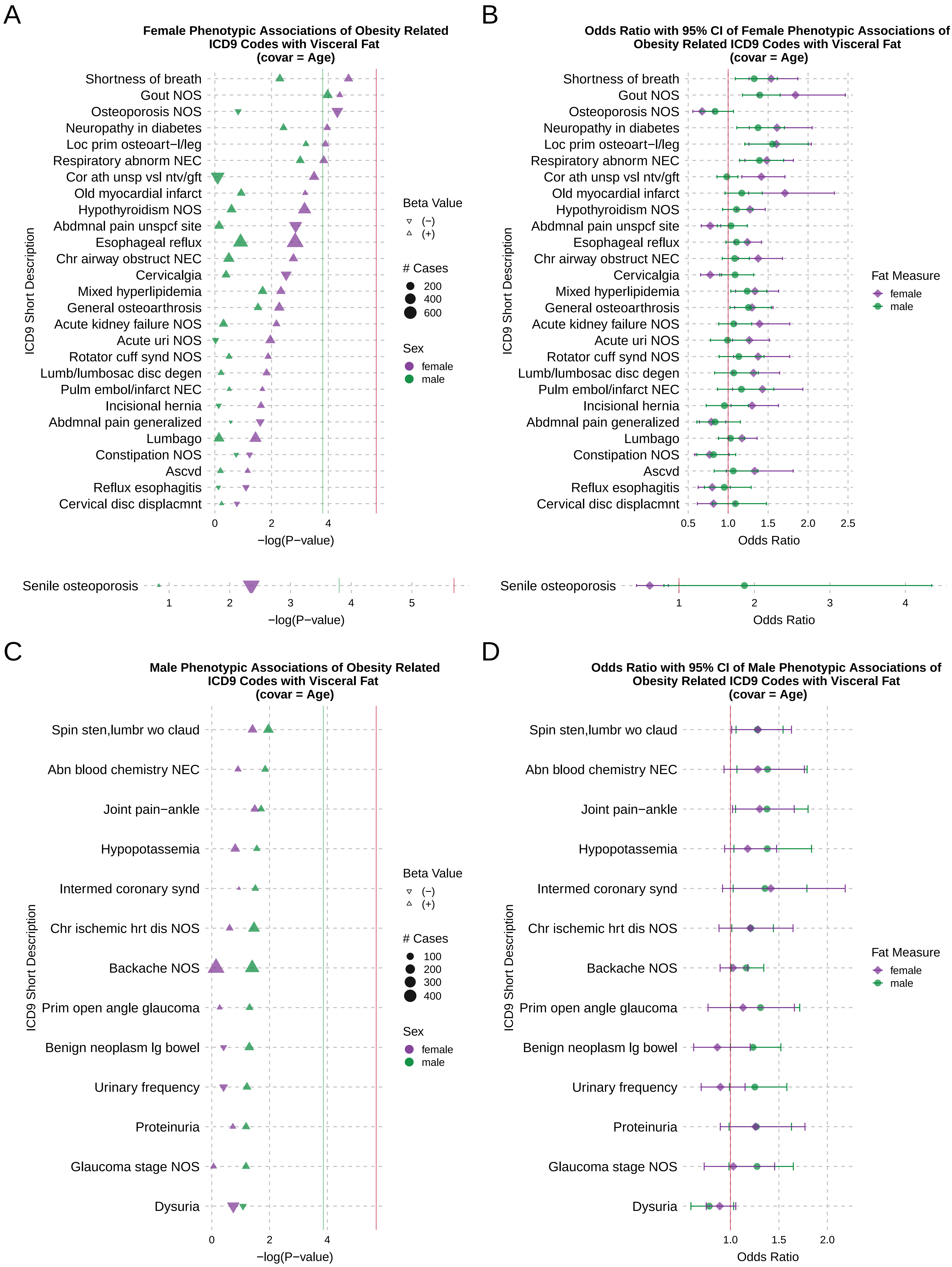
A
Female Phenotypic Associations of Obesity Related ICD9 Codes with Visceral Fat (covar = Age)
ICD9 Short Description
Shortness of breath
Gout NOS
Osteoporosis NOS
Neuropathy in diabetes
Loc prim osteoart−l/leg
Respiratory abnorm NEC
Cor ath unsp vsl ntv/gft
Old myocardial infarct
Hypothyroidism NOS
Abdmnal pain unspcf site
Esophageal reflux
Chr airway obstruct NEC
Cervicalgia
Mixed hyperlipidemia
General osteoarthrosis
Acute kidney failure NOS
Acute uri NOS
Rotator cuff synd NOS
Lumb/lumbosac disc degen
Pulm embol/infarct NEC
Incisional hernia
Abdmnal pain generalized
Lumbago
Constipation NOS
Ascvd
Reflux esophagitis
Cervical disc displacmnt
0 2 4
−log(P−value)
Beta Value
(−)
(+)
Cases
200
400
600
Sex
female
male
Senile osteoporosis
1 2 3 4 5
−log(P−value)
B
Odds Ratio with 95% CI of Female Phenotypic Associations of Obesity Related ICD9 Codes with Visceral Fat (covar = Age)
ICD9 Short Description
0.5 1.0 1.5 2.0 2.5
Odds Ratio
Fat Measure
female
male
Senile osteoporosis
1 2 3 4
Odds Ratio
C
Male Phenotypic Associations of Obesity Related ICD9 Codes with Visceral Fat (covar = Age)
ICD9 Short Description
Spin sten,lumbr wo claud
Abn blood chemistry NEC
Joint pain−ankle
Hypopotassemia
Intermed coronary synd
Chr ischemic hrt dis NOS
Backache NOS
Prim open angle glaucoma
Benign neoplasm lg bowel
Urinary frequency
Proteinuria
Glaucoma stage NOS
Dysuria
0 2 4
−log(P−value)
Beta Value
(−)
(+)
Cases
100
200
300
400
Sex
female
male
D
Odds Ratio with 95% CI of Male Phenotypic Associations of Obesity Related ICD9 Codes with Visceral Fat (covar = Age)
ICD9 Short Description
1.0 1.5 2.0
Odds Ratio
Fat Measure
female
male
