# Supplementary figures and images for: Using Adipose Measures from Health Care Provider-Based Imaging Data for Discovery
Source: J Obes. 2018 Sep 27;2018:3253096. doi: 10.1155/2018/3253096 (PMC6180992; doi:10.1155/2018/3253096)

Phenotypic Associations of ICD9 Codes with Visceral and Subcutaneous Fat  
(covar = Age/Sex)

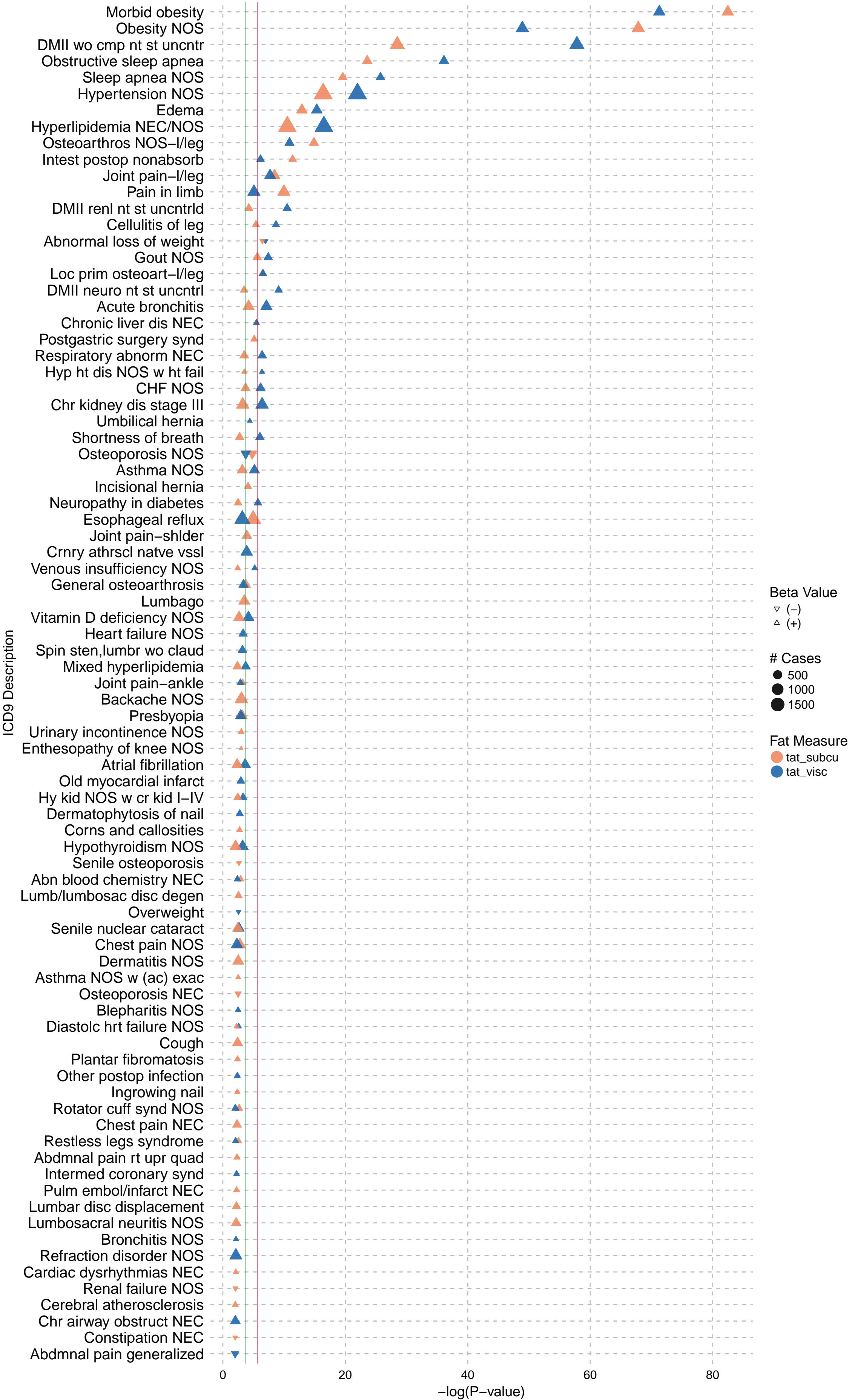

Supplement: Supplementary Materials — Supplementary Figure 1: the plot shows −log(p-values) for all phenotypic associations between ICD-9-based diagnoses on the y-axis for both VAT and SAT after controlling for age and sex. Point size is indicative of the number of cases (500, 1000, and 1500), and the direction of the point, upwards or downwards, represents the direction of the beta estimate (positive or negative). Supplementary Figure 2: the plot shows −log(p-values) for sex-stratified phenotypic associations, outside of the top results, of ICD-9-based diagnoses (y-axis) associated with SAT after controlling for age. (A) Results of female phenotypic associations of ICD-9 codes with SAT, after controlling for age, which show a stronger relationship for females compared to males. (B) Results of odds ratios and 95% CIs of female phenotypic associations of ICD-9 codes with SAT after controlling for age. (C) Results of male phenotypic associations of ICD-9 codes with SAT, after controlling for age, which show a stronger relationship for males compared to females. (D) Results of odds ratios and 95% CIs of male phenotypic associations of ICD-9 codes with SAT after controlling for age. Supplementary Figure 3: the plot shows −log(p-values) for sex-stratified phenotypic associations, outside of the top results, of ICD-9-based diagnoses (y-axis) associated with VAT after controlling for age. (A) Results of female phenotypic associations of ICD-9 codes with VAT, after controlling for age, which show a stronger relationship for females compared to males. (B) Results of odds ratios and 95% CIs of female phenotypic associations of ICD-9 codes with VAT after controlling for age. (C) Results of male phenotypic associations of ICD-9 codes with VAT, after controlling for age, which show a stronger relationship for males compared to females. (D) Results of odds ratios and 95% CIs of male phenotypic associations of ICD-9 codes with VAT after controlling for age. Supplementary Figure 4: Manhattan plots showing results for s [file 3253096.f1.zip › 3253096.f1/Supplementary Figure 1_JOBE_2418542.pdf]
